# Cognitively controlled timing and executive functions develop in parallel? A glimpse on childhood research

**DOI:** 10.3389/fnbeh.2013.00146

**Published:** 2013-10-10

**Authors:** Carmelo M. Vicario

**Affiliations:** School of Psychology, University of QueenslandBrisbane, QLD, Australia

**Keywords:** executive functions, time processing, childhood development, attention, working memory, impulsivity control

Accurate temporal estimations are essential in order to face the surrounding variety of everyday situations (Vicario et al., 2013a). Executive functions (EF) seem strongly involved in timing ability, allowing us to codify temporal intervals, reproduce durations and/or re-call them after a previous encoding phase. In particular, time processing abilities seem related to three different domains of our EF such as working memory (WM) (Fortin and Breton, 1995; Fortin and Rousseau, 1998; Mangels et al., 1998; Lewis and Miall, 2006) attention (Rose and Summers, 1995; Casini and Ivry, 1999; Enns et al., 1999; Tse et al., 2004; Brown, 2006; Vicario et al., 2007, 2009, 2011a,b; Vicario, 2011), and impulsivity control (Reynolds and Schiffbauer, 2004; Wittmann and Paulus, 2008; Rubia et al., 2009).

The evidence in support of these relationship is provided not only by the empirical demonstration that the interference with the processing of one of these three EF affects timing performance (e.g., patients with attention or WM deficits are less accurate in time keeping functions. For instance see the works of Casini and Ivry (1999) and Mangels et al. (1998) on patients with prefrontal lesions) but also in theoretical models which explain how the brain keeps memory of time. For example, the pacemaker–accumulator model (Buhusi and Meck, 2009), assumes that the human brain has its own internal clock with a pacemaker producing subjective time units (Zakay and Block, 1997). Wittmann and Paulus (2008) argue a possible influence of impulsivity on the subjective time keeping functions. In fact, it has been suggested that impulsivity might influence the pacemaker rate of this internal clock and therefore the number of accumulated pulses for temporal units (see Wittmann and Paulus, 2008 for a review on the argument).

In this article I expand upon this idea by providing evidence in support of the suggestion that the ability in performing cognitively controlled timing tasks develop in parallel with these three domains of the EF. This hypothesis basically stems from two arguments: (i) The evidence of a close relationship, in childhood populations, between temporal accuracy and the performance in tasks involving WM, attention and impulsivity control; (ii) The evidence of age related functional differences comparing the activity of the prefrontal cortex during the execution of timing as well as WM, attentive and impulsivity control tasks.

The implications behind this hypothesis are intriguing because they may help to clarify, through the study of cognitive development models, the relationship between the development of the EF and the progression of the level of sophistication of time keeping skills. Moreover, the study of the time keeping functions in childhood populations could represent a potential element of evaluation to qualitatively determine and/or monitor the EF development during the critical phases of brain growth. Finally, one advantage in charting the developmental trajectory of time processing and EF at certain critical moments of development is that this can help to differentiate between experience-dependent versus inborn aspects of time and EF.

## Time keeping and executive functions in adulthood

The literature specialized on time keeping has suggested a general distinction between “*cognitively controlled*” and “*automatic*” timing processes (Lewis and Miall, 2006). Factors such as the temporal scale (sub-seconds vs. supra-second), the task typology (motor vs. not motor) and the type of measurement (continuous vs. intermittent) are considered as (have been considered) the key factors underlying this distinction. Therefore it was asserted (Lewis and Miall, 2006) that a typical automatic timing task involves continuous measurement of a series of predictable sub-second intervals defined by movements; on the other hand, a cognitively controlled timing task requires the explicit orientation of attentional sources toward the duration of stimuli lasting more than one second and characterized by some level of discontinuity (e.g., when timing is broken into discrete measurements by the presence of unpredictable irregular intervals). In reality, this distinction may be more flexible' since cognitively controlled timing tasks may also involve non-motor timing tasks of sub-second durations (e.g., time comparison tasks which require the involvement of decision-making processes—see Vicario, 2013a,b for a complete discussion on this argument) as well as supra-second motor timing tasks (e.g., the classical time reproduction).

In the literature on cognitively controlled timing, several works have provided direct support for the relationship between some EF and time keeping performance. In particular, it has been shown that this function can be influenced by WM, attention and impulsivity/inhibition skills.

Behavioral studies conducted on adult participants have reported that WM and time measurement draw upon the same cognitive resources. For example, it has been shown that secondary tasks involving phonological WM disrupt timing skills (Fortin and Breton, 1995).

Attention manipulation also influences performance in cognitively controlled temporal tasks (Vicario et al., 2007, 2009). For example, it was shown that optokinetic stimulation, which is known to influence spatial attention (Mattingley et al., 1994), affects the participants' performance in temporal decision tasks such as the temporal discrimination of visual stimuli (Vicario et al., 2007).

Finally, evidence of a relationship between time keeping abilities and impulsivity control has been provided by the work of Reynolds and Schiffbauer (2004), which showed that impulsivity due to sleep deprivation causes temporal underestimation in the multiple-seconds range. The recent study by Vicario et al. (2010) on childhood Tourette participants provides a further insight to the link between impulsivity control and time keeping mechanisms. In fact, the authors reported an inverse correlation between temporal accuracy and tic severity scores of these patients. These results might be explained by a compensatory process of neuro-plasticity, which is probably related to the gain of (inhibitory) control over tics through the development of compensatory self-regulation mechanisms.

All the studies support the central role of WM, attention and impulsivity/inhibition skills on cognitively controlled timing tasks. However, we cannot exclude that future investigations may extend the influence of EF on timing skills to other higher-level constructs classified under the umbrella EF term.

## Time keeping and executive functions in childhood

Although there is evidence supporting a very early ability of infants in detecting the temporal features of environmental stimuli (Brackbill and Fitzgerald, 1972), numerous studies have suggested that timing skills improve throughout childhood (see Allman et al., 2012 and Droit-Volet, 2013 for some recent review). For example, the Droit-Volet research team has in several occasions documented that the temporal sensitivity improves with age. By using a time bisection task which makes it possible to calculate a precise index of time sensitivity, namely the Weber ratio, the authors found an improvement with age for both sub-second and supra-second temporal intervals (Droit-Volet and Clement, 2005; Droit-Volet et al., 2008; Zelanti and Droit-Volet, 2011).

Moreover, Chatham et al. (2009) recently found that 3.5 year old fail to use proactive control, which can be interpreted as evidence of a failure to proactively prepare for the predictable future (Shallice and Vallesi, 2007). In fact, proactive control can be considered in relation to time keeping skills, since it mediates the capacity to anticipate and prepare for future events (Chatham et al., 2009). Finally, the recent longitudinal study of Forman et al. (2011) showed that the higher the gain in WM development the better the timing calibration.

Similar age related progressions have been reported for WM, attention, and impulsivity control skills. For example, Hitch and Halliday (1983) and Hulme and Tordoff (1989) have reported that 3–4-year-old children are already capable of retaining information in their phonological store. This provides evidence in support of an early development of WM skills. However, it has been noticed that children cannot perform sub-vocal rehearsal until 7 or 8 years of age; therefore until this time, the information stored in the phonological loop rapidly decays (Gathercole, 2008). This evidence is supported by Gathercole and Alloway (2008), who have found that in Anglo-Saxon participants the digit span increases with age until 15 years. However, a subsequent study on a Spanish population has found that this age limit extends to 17 years (Sebastián and Hernández-Gil, 2012).

Many studies have also demonstrated age-related improvements in selective attention (Trick and Enns, 1998; Scerif et al., 2004), sustained attention (Aylward et al., 2002) and attentional control (Jacques and Zelazo, 2001). For example, Aylward et al. (2002) used the Gordon Diagnostic System (Gordon, 1983) for testing auditory and visual vigilance and the distractibility in a sample of 643 children (Mean age 9.76). The authors found an inverse relationship between error score and the age of participants. A similar age related progression has been documented for impulsivity control, which has been reported to be quite low in children Bjorklund and Harnishfeger (1995). For example, Hughes and Russell (1993) used a ‘day–night' task (Gerstadt et al., 1994) which required children to inhibit a well-established naming response to picture cards. Once again, the authors showed a progressive improvement in this task in children between the ages of 3 and 7 years.

Neuroimaging works provide a further support to the hypothesis that time keeping abilities and executive functions develop in tandem.

In adulthood, there is compelling evidence showing an important role of these regions in timing abilities (Koch et al., 2003; Jones et al., 2004; See Wiener et al., 2010 for review). For instance, Koch et al. (2003) have shown that repetitive Transcranial Magnetic Stimulation upon the right dorsolateral prefrontal cortex (DLPFC) causes temporal underestimation of supra-second durations.

On the other hand, this neural structure is involved in WM (Wager and Smith, 2003), attention (Peers et al., 2013) and impulsivity control (Jasinska, 2013) functions. Moreover, there is evidence documenting a co-existence of timing, WM and attentive deficits in patients with prefrontal lesions (for example see Mangels et al., 1998 and Casini and Ivry, 1999).

A prefrontal activity has been documented even in children while performing a timing task. For instance, the recent study of Smith et al. (2011) has shown an age-related increases in the activation of several regions of the prefrontal lobe, including the DLPFC, while performing a temporal discrimination of supra-second durations (i.e., cognitively controlled timing). In a similar fashion, studies on childhood populations show that the activity of prefrontal regions is influenced by WM, attentive and impulsivity control tasks (Smith et al., 2004; Scherf et al., 2006; Rubia et al., 2006). However, all these works reported a pattern of underactivation during the execution of the above mentioned tasks. Interestingly, according to what has been reported in behavioral works, these studies show evidence that the activation of the prefrontal cortex increases with the age of participants. The development of white matter in the prefrontal regions through adolescence (Schmithorst and Yuan, 2010), could be the cause of these changes in the neural activation of this area and the performance in the tasks described above.

## Conclusion

In this short overview I discussed the literature in support of the suggestion that the ability of children in performing cognitively controlled timing tasks develops in parallel to WM, attention and inhibitory control functions. Independent behavioral studies are in support of this assumption by showing the existence of age related performance improvements for all these cognitive functions. These functions have also been put in relationship with the existence of an age related prefrontal cortex activity, which might be presumably due to the white matter increment continuing through adolescence and into adulthood (Schmithorst and Yuan, 2010).

Although it is possible that other dimensions of the EF domain may have an influence on the development and support of time keeping abilities, we can only discuss the relationship between time keeping and EF functions within the limits of the available literature. This implies that cognitively controlled timing skills might develop and take place from the same basic (i.e., neural and cognitive) mechanisms involved in the formation of three dimensions of EF discussed in this article. However, cognitively controlled timing skills cannot be reduced to these three EF, considering that the representation of time is built also with the active involvement of other processes (e.g., those implied in the representation of space and quantity, see Walsh, 2003 and Vicario et al., 2013b for some review) and brain regions (e.g., parietal cortex, see Wiener et al., 2010 for a review) that cannot be directly linked to EF.

Future works devoted to exploring the developmental hypothesis discussed in this paper may wish to combine behavioral measures and brain methods in a longitudinal perspective, which may be recognized as important in addressing the link between cognitive and neural development. This approach would help to clarify whether and how these three domains of EF and cognitively controlled timing skills develop in parallel.
